# Sodium Ion Conductivity in Superionic IL-Impregnated Metal-Organic Frameworks: Enhancing Stability Through Structural Disorder

**DOI:** 10.1038/s41598-020-60198-w

**Published:** 2020-02-26

**Authors:** Vahid Nozari, Courtney Calahoo, Joshua M. Tuffnell, Philipp Adelhelm, Katrin Wondraczek, Siân E. Dutton, Thomas D. Bennett, Lothar Wondraczek

**Affiliations:** 1 Otto Schott Institute of Materials Research, University of Jena, Jena, Germany; 2 Department of Materials Science and Metallurgy, University of Cambridge, Cambridge, United Kingdom; 3 Cavendish Laboratory, Department of Physics, University of Cambridge, Cambridge, CB3 0HE United Kingdom; 4 Institute of Technical and Environmental Chemistry, University of Jena, Jena, Germany; 5 Center of Energy and Environmental Chemistry, University of Jena, Jena, Germany; 6 Leibniz Institute of Photonic Technologies, Jena, Germany

**Keywords:** Materials chemistry, Materials science, Materials for energy and catalysis

## Abstract

Metal-organic frameworks (MOFs) are intriguing host materials in composite electrolytes due to their ability for tailoring host-guest interactions by chemical tuning of the MOF backbone. Here, we introduce particularly high sodium ion conductivity into the zeolitic imidazolate framework ZIF-8 by impregnation with the sodium-salt-containing ionic liquid (IL) (Na_0.1_EMIM_0.9_)TFSI. We demonstrate an ionic conductivity exceeding 2 × 10^−4^ S · cm^−1^ at room temperature, with an activation energy as low as 0.26 eV, *i.e*., the highest reported performance for room temperature Na^+^-related ion conduction in MOF-based composite electrolytes to date. Partial amorphization of the ZIF-backbone by ball-milling results in significant enhancement of the composite stability towards exposure to ambient conditions, up to 20 days. While the introduction of network disorder decelerates IL exudation and interactions with ambient contaminants, the ion conductivity is only marginally affected, decreasing with decreasing crystallinity but still maintaining superionic behavior. This highlights the general importance of 3D networks of interconnected pores for efficient ion conduction in MOF/IL blends, whereas pore symmetry is a less stringent condition.

## Introduction

Crystalline metal-organic frameworks (MOFs) consist of metal nodes as coordination centers and organic linkers which self-assemble to form a three-dimensional network. Chemical tailoring of both the inorganic node and the organic linker enables property design for a wide range of applications such as gas storage, gas separation, catalysis and ion conduction^1,2^. An alternative route to tune the properties of a given MOF is post-synthetic modification, for example, by applying pressure, temperature or other exogenous stimuli^3^. Depending on stimulus intensity, such post-treatment can lead to structural collapse and solid-state amorphization of the framework^4–7^. The formation of amorphous MOFs through solid-solid transitions (or, similarly, through quenching of MOF-liquids) is of particular interest due to the distinct variations in chemical, mechanical and physical properties which can be obtained as a result of structural disorder^8^.

Amorphization of MOFs can be achieved via different techniques, including pressure-induced structural collapse, ball-milling, melt-quenching, hot-pressing, and re-melting^8–10^. Of these, ball-milling, or mechanosynthesis, which can also be used to synthesize crystalline MOFs, is the most universally applicable route. The low minimum shear moduli of MOFs have previously been shown to be responsible for facile collapse of systems such as UiO-66 ([Zr_6_O_4_(OH)_4_(1,4-BDC)_6_], BDC = benzenedicarboxylate)^11^. Using calcein as a model drug incorporated into crystalline UiO-66, it was demonstrated that amorphization via ball-milling leads to delayed release of the guest molecule: the timescale of release was increased from ~2 days in the crystalline structure to one month in the amorphous composite as a result of structural collapse^12^. Here, we investigate how structural collapse can also be used to enhance the stability of composite MOF materials, generated by impregnation of a crystalline MOF with an ionic liquid (IL).

ILs are salts which are liquid at temperatures <100 °C. Similarly to MOFs, ILs are chemically tunable through the choice of constituent cations and anions^13,14^. They have recently been used for post-synthetic modification of MOF structures by infiltration of the crystalline pore-network^15,16^. The resulting composites have been proposed for use in catalysis, gas separation or ion conduction^15^. Thus far, however, most such studies have been focused on proton and Li^+^ ion conduction. For instance, Fujie *et al*.^17^ studied ionic conduction in an IL (1-ethyl-3-methylimidazolium bis(trifluoromethylsulfonyl)imide, [EMIM][TFSI])- incorporated ZIF-8 composite. It was shown that the IL molecules inside of the ZIF-8 pores do not exhibit a phase transition at low temperature, implying that no freezing of the ionic liquid takes place. As a result, this nanoconfinement effect produced a higher ionic conductivity of the composite as compared to the bulk IL at low temperature. Following-up on this work, the same authors investigated lithium ion diffusion in ZIF-8 mediated by an IL-salt mixture of [EMIM][TFSI] and LiTFSI^18^. They reported that Li^+^ diffuses through micropores via the exchange of the solvating TFSI^−^ anions similar to the Grotthuss mechanism in proton conductivity^19,20^. Very recently, Yoshida *et al*.^20^. studied ionic conduction in a mesoporous MOF, PCN-777, [Zr_6_O_4_(OH)_10_(H_2_O)_6_(TATB)_2_] (H_3_TATB: 4,4,4-*s*-triazine-2,4,6-triyl-tribenzoic acid), impregnated with [EMI][N(CN)_2_]. The hybrid showed an ion conductivity of 4.4 × 10^−3^ S⋅cm^−1^ at room temperature with an activation energy of 0.20 eV. The authors showed that superionic conduction in the composite was due to the formation of a bulk-like IL region within the mesopores. On a broader perspective, the strategy of using composite structures for tuning electrical and mechanical properties of electrolytes is followed in different research fields. Solid/liquid composites such as the soggy-sand concept^21^, gel electrolytes^22^, solid/solid composites (e.g. an inorganic fillers dispersed in a polymer matrix^23^, bicontinuous structures^24^, inorganic/inorganic composites^25^ and glass ceramics^26,27^) are important examples – with MOF-based materials being a new contender.

Further reports on Li^+^ ion conductivity in MOF structures include the work of Wang *et al*.^28^. who synthesized a composite by incorporating [EMIM_0.8_Li_0.2_][TFSI] into MOF-525 (Cu). The composite showed ionic conductivity of 3.0 × 10^−4^ S⋅cm^−1^ at room temperature, with a Li transference number of 0.36, *i.e*., higher than the pure IL. Whilst this approach relies on the non-MOF component to introduce lithium ions for conduction, the open metal sites in certain MOF frameworks have also been utilized via post-synthetic modification. By grafting the anionic component of a lithium salt directly onto an unsaturated metal center, the lithium ion is free to conduct. For example, LiO*t*Bu-grafted UiO-66 exhibits room temperature ionic conductivities of 1.8 × 10^−5^ S⋅cm^−1^ and an activation energy of 0.18 eV^29^, and LiClO_4_ (in propylene carbonate) grafted onto HKUST-1 showed a room temperature ionic conductivity of 0.38 mS⋅cm^−1^ and an activation energy of 0.18 eV^30^.

Lithium-ion conduction has been investigated extensively for its importance in electrochemical energy storage^31^. However, uneven distribution on Earth (coupled with the changing geopolitical climate) and increasing demand for lithium in electronic devices, electric vehicles and grid storage, have created concerns for the future of rechargeable lithium ion batteries^32^. As an alternative, electrochemical energy storage systems based on sodium are also considered, although at present with a still much smaller variety of generally suitable material candidates^33^. There have been some early studies regarding Na^+^-related ion conduction in MOFs. Cepeda *et al*.^34^. explored Li^+^ and Na^+^ conduction in {[ScM(*μ*_4_-pmdc)_2_(H_2_O)_2_]⋅solv}_n_ [EHU1(Sc,M)] (where M = Li, Na; pmdc = pyrimidine-4,6-dicarboxylate; solv = corresponding solvent). The corresponding Li^+^ and Na^+^ conductivity was 3.8 × 10^−7^ and 1.1 × 10^−7^ S⋅cm^−1^, respectively. Recently, Na^+^-ion conduction was also examined in an anionic Cu-azolate MOF, MIT-20 which was reported with a Na^+^-ion conductivity of 1.8 × 10^−5^ S⋅cm^−1^ at room temperature^35^. One of the issues impeding the application of such electrolytes in real-world devices is the lack of stability outside of inert atmospheres^36^. Here, we speculate that this problem can be addressed by hindering the interactions of secondary guest molecules (*i.e*., originating from the surrounding atmosphere) with the composite by amorphizing the MOF framework and, thus, trapping the IL molecules inside the pores of the amorphous system; however with consideration of a possible trade-off with performance.

Starting from the above hypothesis, we address the two major subjects of achieving enhanced ion conductivity and, at the same time, enhanced stability by IL infiltration of a MOF and subsequent amorphization so as to obtain a highly conductive amorphous composite. For this, we started with incorporating an imidazolium-based IL, 1-ethyl-3-methylimidazolium bis(trifluoromethylsulfonyl)imide, [EMIM][TFSI], containing its corresponding sodium salt [Na][TFSI] into crystalline ZIF-8. This composite (S-IL@ZIF-8) was demonstrated to exhibit superionic properties. Subsequent partial amorphization of the composite using ball-milling lead to significantly enhanced stability under an ambient atmosphere as compared to the crystalline counterpart.

## Results and Discussion

**Figure 1:**
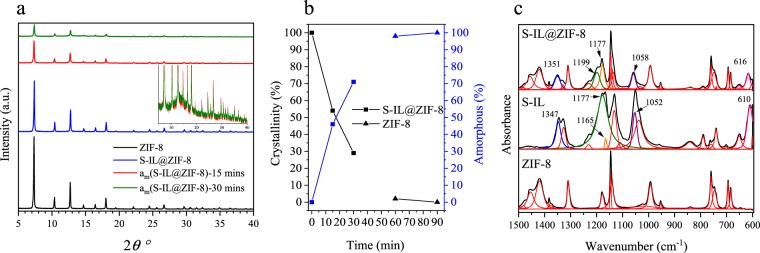
(**a**) XRD patterns of the pristine ZIF-8, S-IL@ZIF-8 composite, and S-IL@ZIF-8 composites ball-milled for fifteen and thirty minutes, respectively. Inset show highlighted regions of XRD spectra for a_m_(S-IL@ZIF-8)-15 mins and a_m_(S-IL@ZIF-8)-30 mins samples. (**b**) Quantification of crystallinity and amorphous fractions as a function of ball-milling time using Rietveld-refinement. Square and triangle symbols represent S-IL@ZIF-8 composite and pristine ZIF-8, respectively. (**c**) FTIR spectra of pristine ZIF-8, S-IL mixture and S-IL@ZIF-8 composite. The spectral resolution is 2 cm^−1^. Shifted peaks in S-IL@ZIF-8 compared to S-IL mixture spectra are highlighted in different colors. Peak deconvolution was performed using a Voigt function in Fityk software^59^.

Crystallinities and morphologies of the pristine ZIF-8 and S-IL@ZIF-8 composites were investigated using XRD and SEM (Figs. 1a and S1). The small variations in peak intensities between the pristine ZIF-8 and ZIF-8 after S-IL incorporation are attributed to confinement of S-IL in the ZIF-8 pores, as has previously been shown in the literature^18^. SEM images confirm that the morphology of the ZIF-8 crystals remains intact after S-IL incorporation (Fig. S1**)**, with FTIR spectra confirming the presence of the S-IL within the composite (Fig. 1c**)**. We find that some vibrational features in S-IL were shifted to higher frequency upon incorporation into ZIF-8: peaks located at 610, 1052, 1165, 1177, and 1347 cm^−1^ which are assigned to SO_2_ antisymmetric bending, SNS antisymmetric stretching, (N)CH_2_ and (N)CH_3_CN stretching, CF_3_ antisymmetric stretching, and SO_2_ antisymmetric stretching are shifted to 616, 1058, 1177, 1199, and 1351 cm^−1^, respectively^37^. As shown in previous studies reporting on incorporation of different ILs in MOFs^38–42^, these distinct shifts in peak positions confirm the successful confinement of S-IL mixture inside ZIF-8.

Both BET surface area and pore volume (Fig. S2 and Table S1) of ZIF-8 decreased significantly after S-IL confinement (from 1297.3 m^2^ g^−1^ and 0.641 cc STP g^−1^ respectively in ZIF-8, to 7.29 m^2^ g^−1^ and 0.006 cc STP g^−1^ respectively in S-IL@ZIF-8), indicating that the S-IL solution occupies the pores in ZIF-8^43,44^. Since the thermal stability limit of such composites determines the potential application of the electrolytes in the desired operation conditions, thermogravimetric analysis was conducted and the corresponding decomposition temperatures were measured (Fig. S3a and Table S2**)**. According to the onset temperatures of decomposition, the S-IL@ZIF-8 composite starts decomposing at a lower temperature (388 °C) compared to bulk S-IL (423 °C). This is a small decrease, and the high thermal stability of the composite remains suitable for applications in electrochemical energy storage devices. In good agreement with the IR peak shifts, the lower decomposition temperature for S-IL@MOF is attributed to the immobilizing interaction between IL molecules and the MOF framework^44,45^. Consistent with this result, DSC profiles (Fig. S3b) contain no evidence of any phase transition occurring in the S-IL@ZIF-8 composite up to the decomposition temperature (388 °C). The DSC signal at temperatures above 400 °C is attributed to thermal decomposition of the samples.

**Figure 2:**
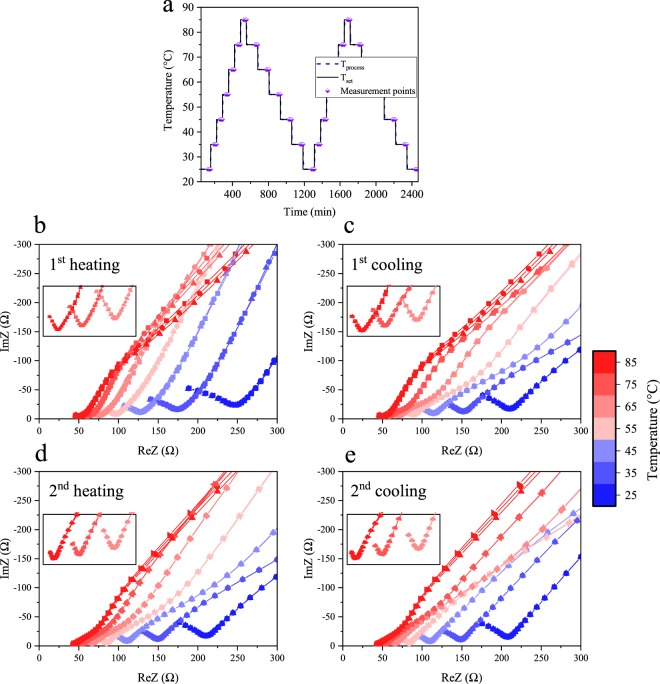
Thermal sweep AC impedance measurements of S-IL@ZIF-8 composite. (**a**) Temperature-programmed thermal sweep protocol for impedance measurements of S-IL@ZIF-8 composite. Purple diamonds with lower half filled indicate the isothermal, three fifteen-minutes spaced runs at each equilibrated temperature step. (**b–e**) Nyquist plots of each heating and cooling cycle, where the blue to red transition indicates increasing the temperature from 25 °C to 85 °C with 10 °C increments. At each temperature step, the first, second and third run is indicated with square, circle and triangle symbols, respectively. The error bars are too small to be seen in this scale; corresponding error bars are shown in Fig. S4 at different scaling. Insets in Fig. 2b–e show semicircles above 55 °C. The solid lines are a guide for the eye.

Using alternating current (AC) electrical impedance spectroscopy, the ionic conductivity of the S-IL@ZIF-8 composite was investigated using the thermal sweep protocol depicted in Fig. 2a (*see* also Methods section). The obtained Nyquist plots for the S-IL@ZIF-8 composite show the typical behavior expected for ionic conductors: a semicircle arched upwards at higher frequency and a tail in the low frequency region (Fig. 2b–e)^34,46,47^. The error bars in Nyquist plots according to instrument accuracy are depicted for each frequency point in Fig. S4. Ionic resistance values were extracted from the data by taking the intersection between the semicircle and the tail, as reported previously^30^. We found the difference between the typical method for calculating the resistance, fitting the semicircle with a circle function, to differ from taking the intersection by less than four percent.

**Figure 3:**
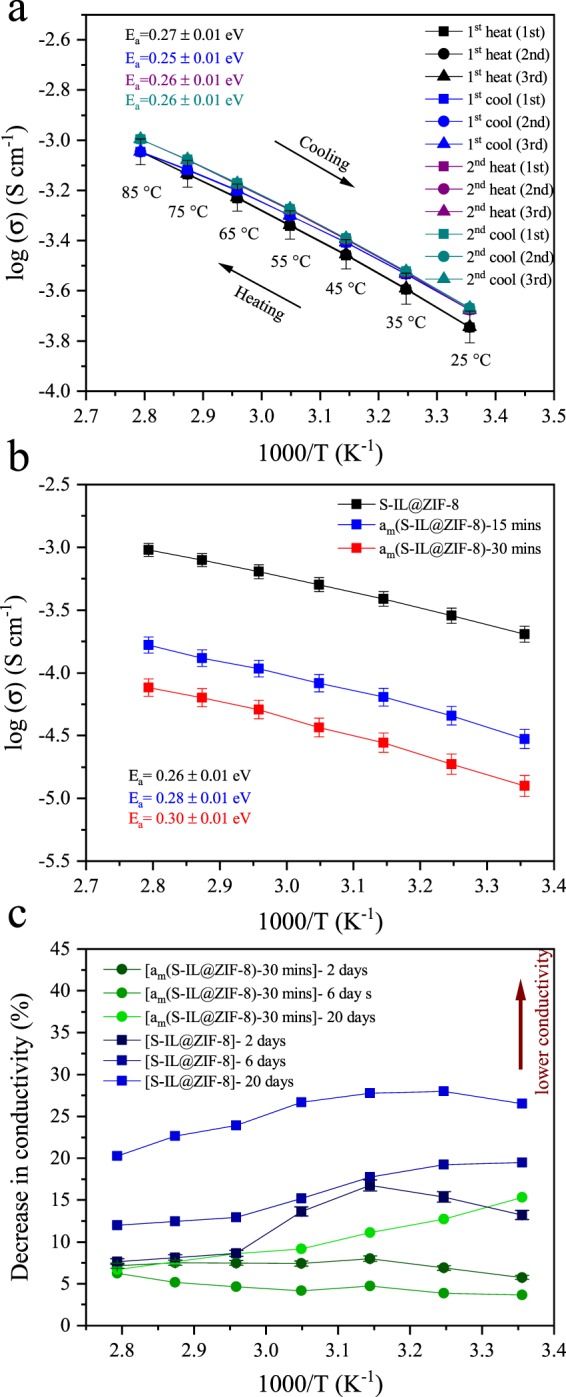
Arrhenius plots of S-IL@ZIF-8 composite. (**a**) Arrhenius plots of S-IL@ZIF-8 of heating and cooling cycles. At each heating and cooling step, three independent runs with fifteen-minute intervals were performed, shown as square, circle and triangle symbols. Second heating and cooling cycles overlap each other. (**b**) Arrhenius plots of S-IL@ZIF-8 composite ball-milled for fifteen and thirty minutes. (**c**) Change in the ionic conductivity of crystalline (squares), S-IL@ZIF-8, and amorphized (circles), a_m_(S-IL@ZIF-8)-30 mins, composites upon exposing the samples to ambient atmosphere for two, six, and twenty days. Error bars are in the range of four percent.

For solid state electrolyte applications, the importance of sample stability over multiple thermal sweeps and repetitive measurements is paramount. Ionic conductivities of each heating and cooling cycle are very similar, with only a slight increase in conductivity observed upon first heating cycle. This may be attributed to the thermal relaxation of the composite in pellet form during the first heating cycle. The second heating and cooling cycles overlap completely. Nyquist plots of the three isothermal consecutive measurements at each temperature step (Fig. 2b–e) and the corresponding Arrhenius plots (Fig. 3a) of the heating and cooling cycles validate that there is no hysteresis and the composite is stable upon heating and cooling cycles with multiple measurements at each step.

From the slope of the Arrhenius plots we derived activation energies for each cycle. The activation energy for the first heating cycle is slightly higher than that of the other cycles, most likely originating from thermal relaxation effects. The average value of the activation energy for the S-IL@ZIF-8 composite is 0.26 eV, which is among the lowest values observed for MOF-based ionic conductors^34,35,46^. Together with the ZIF-8 pore aperture (~3.4 Å)^48^, the observed value of activation energy suggests that Na^+^ ions are conducting through micropores similar to a Grotthuss mechanism by exchanging the solvating TFSI^−^ anions (we note that coordination environment in the bulk S-IL is [Na(TFSI)_3_]^2−^)^49^. Similar observations have been made previously on Li^+^ migration in ZIF-8^18^. The composite exhibits an ionic conductivity of ~2×10^−4^ S ⋅cm^−1^ at room temperature. To the best of our knowledge, this is the highest reported value in the literature for Na^+^-related ion conduction in MOF-based composite electrolytes so far^34,35^. Such low activation energy and high conductivity classify the composite as a superionic conductor^50^.

An independent high temperature thermal sweep impedance measurement was carried out in a separate laboratory (in University of Cambridge) within the temperature range of 25 °C to 125 °C with 20 °C increments (Fig. S5). Very good reproducibility was observed for the activation energy value (0.26 eV).

The instability of composites or other types of electrolytes in humidity or ambient air is a challenging issue, as it places a limit on the applicability outside of inert atmospheres. Here, we address this subject by partially amorphizing the MOF framework via ball-milling of the crystalline S-IL@ZIF-8 composite. In doing so, we aim for enhanced material stability while maintaining the ionic conducting performance^7,51–54^. Ball-milling was performed on separate batches of S-IL@ZIF-8 composites under an inert atmosphere for fifteen to ninety minutes. However, as a result of gradually reducing pore volume, the IL was partially expelled from the composite upon ball-milling for 60 and 90 minutes, thus, we focus only on the partially amorphized samples of a_m_(S-IL@ZIF-8)-15 mins and a_m_(S-IL@ZIF-8)-30 mins. During ball-milling, the particle size was observed to decrease (Fig. S1). Most notably, crystallinity was observed to decrease to 29% and 54% (see Methods section) for a_m_(S-IL@ZIF-8)-30 mins and a_m_(S-IL@ZIF-8)-15 mins, respectively (Figs. 1, S1), differentiated from the effect of particle size by the progressive rise in diffuse scattering observed in the inset of Fig. 1a. Full amorphization of pristine ZIF-8 occurred after 60 and 90 min of ball milling (Fig. 1b). Corresponding SEM micrographs of the amorphized ZIF-8 samples (a_m_(ZIF-8)-60 mins and a_m_(ZIF-8)-90 mins) are provided in Fig. S11. XRD patterns in Fig. S6 compare the stability of pristine ZIF-8 and the S-IL@ZIF-8 composite towards ball-milling: the observed differences between the collapse time of S-IL@ZIF-8 and that of pristine ZIF-8 reported previously are ascribed to the presence of a liquid medium within the MOF pores during ball-milling, which enhances the resistance to structural collapse by ball-milling^7,55^. We can conclude that the presence of IL molecules increases the mechanical stability of pristine ZIF-8.

FTIR spectra of a_m_(S-IL@ZIF-8)-15 mins and a_m_(S-IL@ZIF-8)-30 mins (Fig. S7) confirm that the samples retained their chemical integrity and that, at the same time, the S-IL solution remained inside of the pores upon ball-milling. Corresponding TGA scans revealed similar thermal decomposition as with the crystalline composites. BET surface area and pore volume (Fig. S2 and Table S1) of the a_m_(S-IL@ZIF-8)-15 mins and a_m_(S-IL@ZIF-8)-30 mins were significantly decreased as compared to those of pristine ZIF-8, which is consistent with a previous study on ball-milling of ZIF-8^7^. We note that the BET surface area of the a_m_(S-IL@ZIF-8)-30 min sample slightly exceeds that of a_m_(S-IL@ZIF-8)-15 min; this may originate from the smaller particle size in the a_m_(S-IL@ZIF-8)-30 min sample (*see* Fig. S1).

The results of AC impedance measurements conducted under inert atmosphere on partially amorphized samples are summarized in Fig. 3b. The partially amorphized samples exhibit a somewhat lower ionic conductivity of 2.97 × 10^−5^ S⋅cm^−1^ and 1.26 × 10^−5^ S⋅cm^−1^ for a_m_(S-IL@ZIF-8)-15 mins and a_m_(S-IL@ZIF-8)-30 mins, respectively, as compared to the crystalline composites (2 × 10^−4^ S⋅cm^−1^) at room temperature (Fig. 3b). Also, the activation energy increases slightly from 0.26 eV for S-IL@ZIF-8 to 0.28 and 0.30 eV for a_m_(S-IL@ZIF-8)-15 mins and a_m_(S-IL@ZIF-8)-30 mins, respectively. Both observations indicate that amorphization exerts a disrupting effect on the interconnected conduction channels within the MOF framework.

For evaluating the stability of crystalline S-IL@ZIF-8 in comparison to a_m_(S-IL@ZIF-8)-30 mins, we monitored the ion conductivity by re-measuring after exposure to ambient air (*T* = 20 °C, humidity ~45%) for different periods of time (*i.e*., from 2–20 days). The corresponding Arrhenius plots are presented in Fig. S8, demonstrating the effects of exposure: conductivities for both crystalline and amorphized samples decrease relative to the values of samples which were kept under inert conditions. For the crystalline composite, this decrease appears significant already after two days of exposure, where the conductivity was found to decrease by about 8%, and further by ~20% after 20 days of exposure when measured at 85 °C. The relative change in ionic conductivity is plotted in Fig. 3c after normalizing the difference between ambient and inert atmosphere storage. For the partially amorphized sample under identical storage conditions, the decrease is only 6% after 20 days (when measured at 85 °C). When re-measured at room temperature (25 °C), the decrease in conductivity is more substantial for the crystalline sample (up to one third after 20 days of storage), while the partially amorphized sample shows only 15% decrease even after 20 days of storage. Moreover, the change in activation energies are more significant in crystalline composite compared to the partially amorphous one. For example, in the crystalline composite, the activation energy increases from 0.26 eV to 0.38 eV and 0.4 eV after two and six days of exposure, respectively, whereas the activation energy in the partially amorphous composite remained unchanged after two days and increased slightly to 0.28 eV after six days of exposure (the notable temperature dependence of degradation indicates a certain amount of recovery when re-drying the material). Clearly, partial MOF amorphization provides a powerful tool for enhancing the stability of conduction processes in IL@MOF composites. At the moment, we do not have definite answer as to the mechanism of this effect. However, we infer that amorphization impedes the interaction of guest molecules with the composite which in turn enhances long-term stability.

## Conclusion

In summary, we report on a promising composite electrolyte via encapsulation of an IL into a crystalline MOF (ZIF-8), showing very high sodium ion conduction with low activation energy. We investigated the effect of structural amorphization on the ionic conductivity of this emerging class of collapsed MOF composites. Partially amorphized MOFs exhibit notably enhanced stability in terms of persistence of high ionic conductivity under ambient conditions as compared to their crystalline counterparts. This provides a novel tool for tailoring the functionality of MOF composites by generating structural disorder; in particular, a major shortcoming of many MOF-based materials can be addressed in this way while keeping the advantages of functionalization. This ‘*best of both worlds*’ situation expands the possible applications for MOFs in which crystalline composites may have serious drawbacks.

## Methods

### Preparation of S-IL@ZIF-8 composites

The IL, 1-ethyl-3-methylimidazolium bis(trifluoromethylsulfonyl)imide, [EMIM][TFSI] (>99%) and its corresponding sodium salt, sodium bis (trifluoromethylsulfonyl)imide, [Na][TFSI] (99.5%), were purchased from IoLiTec and Solvionic, respectively, and used as received. Water contents of the IL and salt were measured using Karl-Fischer titration and found to be less than 20 ppm. ZIF-8 was purchased from ACSYNAM Inc. All the compounds were stored inside an Ar-filled glovebox upon arrival, with O_2_ and H_2_O levels of less than 0.1 ppm. Because of the viscosity increase upon dissolving more salt in the IL, salt-IL (S-IL) solutions were prepared by dissolving 10 mol% of salt in its corresponding IL. The mixture was stirred overnight at 70 °C to obtain a fully dissolved and clear S-IL solution (with three TFSI^−^ coordinated to each Na^+^ in the S-IL system)^49^. ZIF-8 was evacuated at 125 °C under vacuum overnight prior to use in order to remove moisture and other impurities. The S-IL@ZIF-8 composite loaded with 35 wt% S-IL solution (*i.e*., the maximum loading to obtain the composite in powder form) with ionic conductivity of 6 × 10^−3^ S ⋅cm^−1^ at 25 °C was prepared using the capillary action method^49,56^. The theoretical volume occupancy of S-IL from S-IL density (1.54 g cm^−3^)^49^ and ZIF-8 pore volume (0.64 cm³ g^−1^) was 55%. Based on the number of supercages per mol of ZIF-8 (1.0 × 10^23^ cages mol^−1^)^43^, the number of S-IL in each cage was calculated to be 1.89 on average. The S-IL solution was added dropwise into ZIF-8 and mixed thoroughly using mortar and pestle to obtain homogeneous powder samples. This procedure was repeated for several times until the whole S-IL mixture was added to ZIF-8. Preparation of S-IL@ZIF-8 composite took around one hour. To enhance the diffusion of S-IL solution into ZIF-8 pores, the as-prepared composite was kept at 80 °C overnight^18^. All synthesis and sample preparation steps were performed inside an Ar-filled glovebox to prevent water adsorption on the salt, on the IL or on the S-IL@ZIF-8 composite.

### X-ray diffraction (XRD)

X-ray diffractograms were collected using a Rigaku SmartLab diffractometer (Cu K_α_ X-ray source with wavelength of 1.54059 Å) with a HyPix-3000 (horizontal configuration) detector in 1D scanning mode. The voltage and current of the X-ray tube were set to 40 kV and 50 mA, respectively. General Bragg-Brentano geometry was employed with a 10 mm length-limiting slit at incident section and a 2.5° Soller slit with a K_β_ filter in receiving part. The diffraction patterns were obtained in the 2Θ range of 5 to 50° with step size of 0.01° at a rate of 10°·min^−1^. Rietveld- refinement^57^ was performed to quantify the crystalline and amorphous phases in ball-milled samples, using the MAUD^58^ software package. The LaB_6_ diffractogram was selected for reference.

### Thermogravimetric analysis (TGA)

A Netzsch STA 449 F1 instrument was used for TGA and differential scanning calorimetry (DSC) analysis. Approximately 10 mg of each sample were placed in a platinum crucible; measurements were performed under 20 ml·min^−1^ nitrogen flow. First, the samples were heated up to 120 °C with a ramp of 20 °C·min^−1^ and equilibrated for eight hours to remove any volatiles. Subsequently, the samples were heated up to 700 °C at a rate of 10 °C·min^−1^.

### Fourier transform infrared (FTIR) spectroscopy

FTIR spectra were collected for the pristine ZIF-8, [EMIM][TFSI], [Na][TFSI], as well as the crystalline and amorphized S-IL@ZIF-8 composites using a Thermo Scientific Nicolet iS10 model FTIR spectrometer equipped with an attenuated total reflection mode. Background (64 scans) and sample (128 scans) spectra were measured with a resolution of 2 cm^−1^. The Fityk software was used to evaluate the collected spectra^59^.

### Brunauer-emmet-teller (BET) analysis

An Autosorb iQ instrument from Quantachrome Instruments was used for BET surface area and pore volume analysis. N_2_ adsorption at 77 K was carried-out to quantify the BET surface area of the samples. Around 50 mg of each sample were loaded into a 9 mm diameter cell inside a glovebox, sealed from atmosphere and installed on to the instrument. Prior to measurement, the samples were outgassed for 20 h under high vacuum (10^−8^ mbar) at 125 °C to remove any kind of impurities from the sample.

### Scanning electron microscopy (SEM)

The morphology of the pristine ZIF-8 as well as of the crystalline and amorphous S-IL@ZIF-8 composites was analyzed using a JSM-7001F microscope (Jeol Ltd, Japan). Approximately 10 mg of each sample were placed on a carbon tape pasted on a cell. The working distance for all samples was set to 15 mm. Samples were coated with a thin layer of carbon before measurements.

### Ball-milling amorphization

Amorphization of the S-IL@ZIF-8 composite was performed using a Retsch PM 100 planetary ball mill. For each ball-milling run, around 1000 mg of sample with forty grinding balls of 5 mm in diameter were placed in a 50 ml jar. The jar and grinding balls were stored in the glovebox one day prior to tests, then, the samples were loaded and sealed using clamps inside the glovebox. The instrument was set to 650 rpm with one-minute intervals during the 15, 30, 60 and 90 minutes of runs. After milling, amorphized samples were recovered inside the glovebox and stored in sealed containers. The corresponding samples were referred to as a_m_(S-IL@ZIF-8)-15 mins and a_m_(S-IL@ZIF-8)-30 mins, respectively.

### Ionic conductivity measurements

A Novocontrol Alpha-A Analyzer was used to carry-out AC impedance measurements in the frequency range of 10^−1^ to 10^7^ Hz^60^. Approximately 450 mg of powder sample were pressed into a pellet of 1.4 mm thickness and 20 mm in diameter by applying 3 tons of pressure load for one minute inside an Ar-filled glovebox. The pellet was placed and sealed in a BDS 1308 sample holder with gold-plated electrodes (Novocontrol Technologies). Thermal sweep tests were performed for two heating and cooling cycles between 25 °C and 85 °C with 10 °C increments and isothermal dwell times, see Fig. 2a. To ensure thermal equilibration within the sample and instrument chamber prior to any measurement, each temperature change was followed by an isothermal hold period with a duration of thirty minutes in case of heating and ninety minutes in case of cooling. At each equilibrated temperature step three consecutive runs of impedance measurement were performed with a fifteen-minute interval between each run. Air-stability tests were performed in the same way after exposure of the crystalline and amorphized samples to ambient atmosphere for two, six and twenty days. Ionic conductivities were determined using the following equation, which considers all of the mobile ionic species.

$$\sigma =(\frac{1}{{R}_{DC}})(\frac{l}{A}),$$

where *R*_DC_ was calculated at the intersection point between the high frequency semi-circle and the low frequency tail in *Nyquist* plots (−Z″ vs. Z′)^30^. *l*/*A* is the geometric ratio between sample thickness *l* and electrode area *A*. The activation energy *E*_A_ was determined from the Arrhenius plot of log (*σT*) versus (1/*T*) accordingly:

$$\sigma T={\sigma }_{0}exp(\frac{{E}_{{\rm{A}}}}{{k}_{{\rm{B}}}T})$$

where *k*_B_ is Boltzmann’s constant^60^.

## Supplementary information

Supplementary Information.
