# Supplementary material for: Sodium Ion Conductivity in Superionic IL-Impregnated Metal-Organic Frameworks: Enhancing Stability Through Structural Disorder
Source: Sci Rep. 2020 Feb 26;10:3532. doi: 10.1038/s41598-020-60198-w (PMC7044296; doi:10.1038/s41598-020-60198-w)
Supplement: Supplementary file 1 — Supplementary Information. [file 41598_2020_60198_MOESM1_ESM.docx]

**Supplementary material**

**for**

**Sodium Ion Conductivity in Superionic IL-Impregnated Metal-Organic Frameworks: Enhancing Stability Through Structural Disorder**

Vahid Nozari^1^, Courtney Calahoo^1^, Joshua M. Tuffnell^2,3^, Philipp Adelhelm^4,5^, Katrin Wondraczek^6^, Sian E. Dutton^3^, Thomas D. Bennett^2^ and Lothar Wondraczek^1,5,*^

^1^*Otto Schott Institute of Materials Research, University of Jena, Jena, Germany*

^2^*Department of Materials Science and Metallurgy, University of Cambridge, United Kingdom*

^3^*Cavendish Laboratory, Department of Physics, University of Cambridge, United Kingdom*

^4^*Institute of Technical and Environmental Chemistry, University of Jena, Jena, Germany*

^5^*Center of Energy and Environmental Chemistry, University of Jena, Germany*

^6^*Leibniz Institute of Photonic Technologies, Jena, Germany*


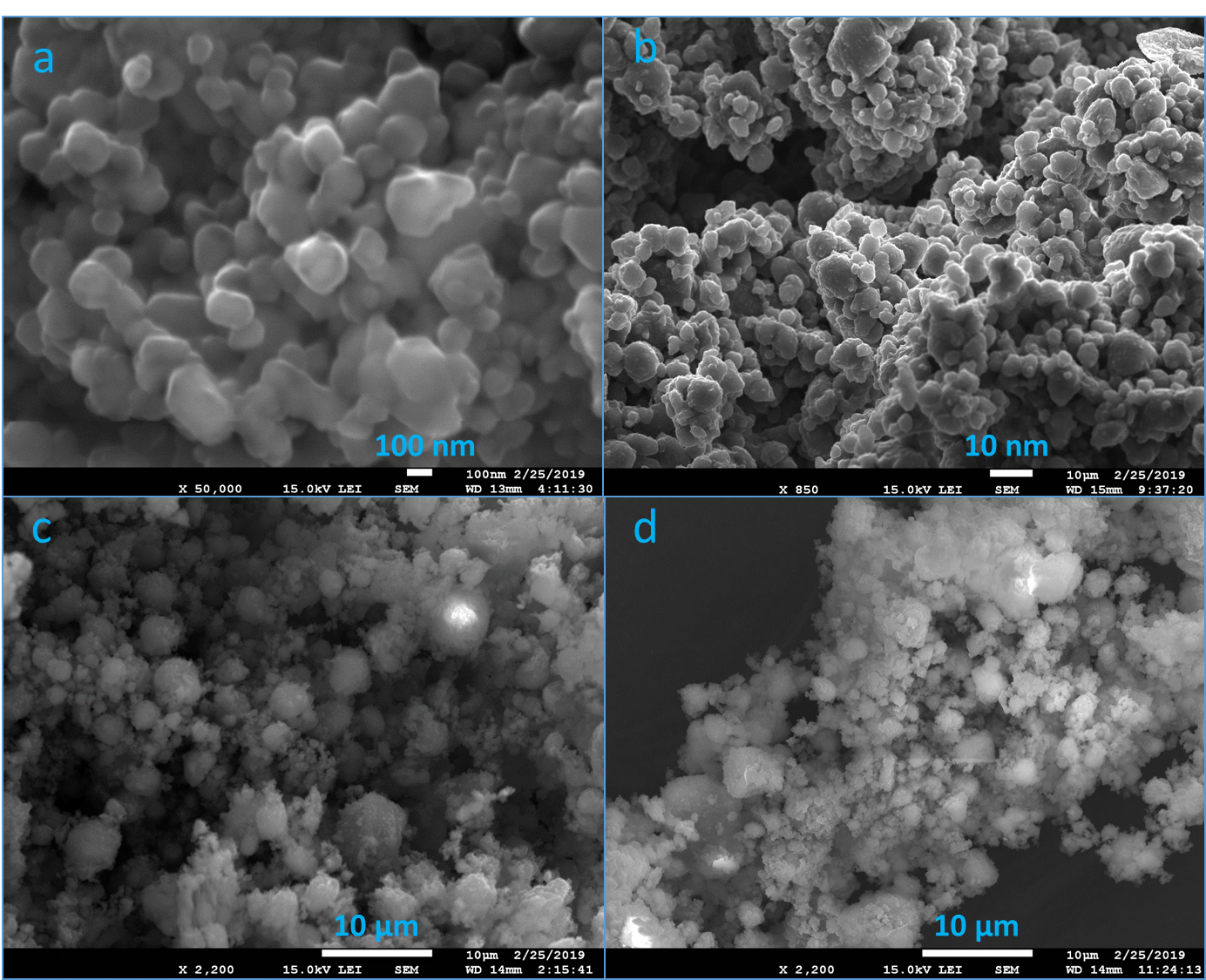


**Figure S1.** SEM images of (**a**) ZIF-8; (**b**) S-IL@ZIF-8; (**c**) a_m_(S-IL@ZIF-8)-15 mins and (**d**) a_m_(S-IL@ZIF-8)-30 mins.

**Figure S2.** N_2_ gas adsorption-desorption measured on pristine ZIF-8, S-IL@ZIF-8 and amorphized samples at 77 K. Empty symbols represent the desorption part. Lines are drawn to guide the eye.

**Table S1.** BET surface area and pore volume analysis. Instrumental error range is within four percent.

| **Sample** | **BET surface area**  **(m^2^/g)** | **DFT pore volume**  **(cc STP/g)** |
| --- | --- | --- |
| ZIF-8 | 1297 | 0.641 |
| S-IL@ZIF-8 | 7.29 | 0.006 |
| a_m_(S-IL@ZIF-8)-15 mins | 11.99 | 0.048 |
| a_m_(S-IL@ZIF-8)-30 mins | 12.03 | 0.049 |

**Figure S3.** (**a**)Thermogravimetric analysis (TGA) and (**b**) Differential scanning calorimetry (DSC) curves obtained with a heating rate of 10 °C min^-1^ under nitrogen flow of 20 ml min^-1^.

**Table S2.** Onset temperatures T_onset_ and decomposition temperatures T_decomp_ for various samples. The onset temperature is defined as the temperature at which the sample has lost two percent of its initial mass. Decomposition temperatures are obtained from intersection of two tangent lines from horizontal and vertical parts of the TGA curves.

| Sample | T_onset_ (°C) | T_decomp_ (°C) |
| --- | --- | --- |
| ZIF-8 | 543 | 613 |
| S (salt) | 400 | 421 |
| IL | 411 | 444 |
| S-IL | 423 | 444 |
| S-IL@ZIF-8 | 388 | 428 |
| a_m_(S-IL@ZIF-8)-15 mins | 388 | 435 |
| a_m_(S-IL@ZIF-8)-30 mins | 373 | 435 |


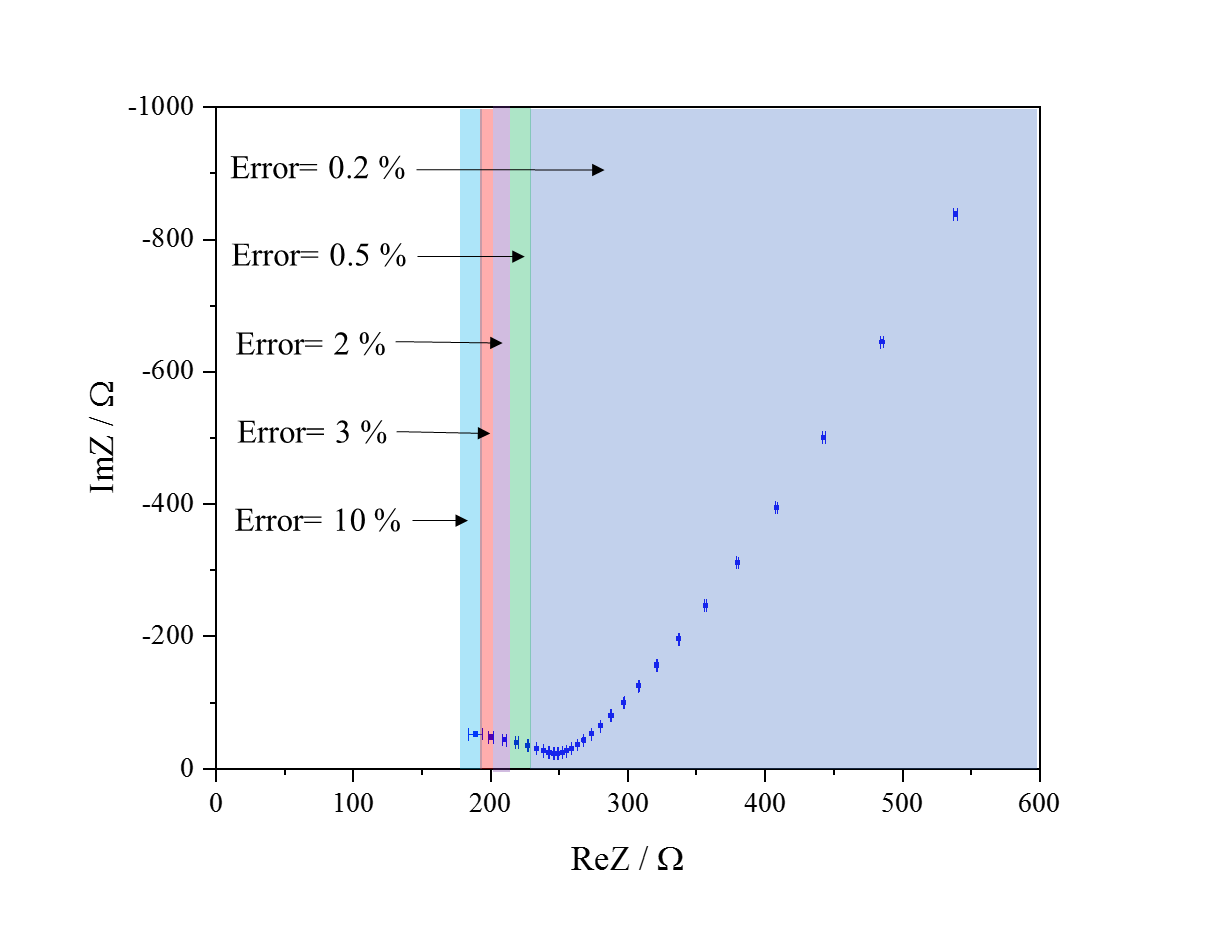


**Figure S4.** Error propagation of the impedance measurements based on instrumental error. At each data point, depending on its individual frequency and impedance values, the error bars are were calculated. The error bars start from 0.2 % in low frequency region to 10 % at the highest frequency value.


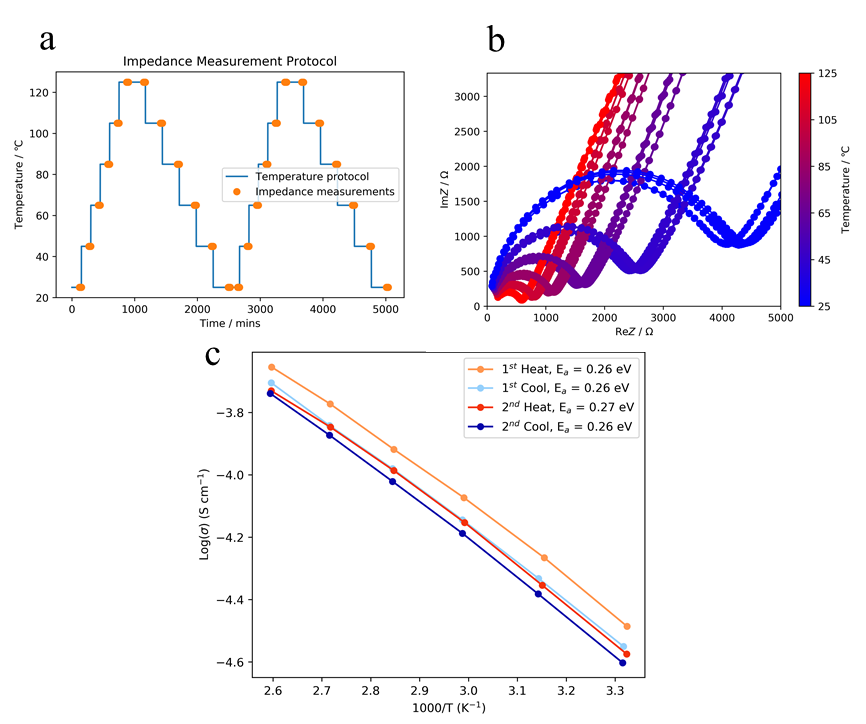


**Figure S5.** Variable temperature AC impedance measurement of S-IL@ZIF-8 on a different lab on a similar setup showing: (**a**) the thermal sweep protocol of two heating and cooling cycles with long thermal equilibration times (blue line) and indication of the three conductivity measurements at each temperature step (orange points); (**b**) Nyquist plots at each temperature step (circles; lines are drawn to guide the eyes) where the color transition from blue to red represents the increasing temperature from 25 °C to 125 °C in 20 °C increments (only the second up and down temperature sweeps are shown for clarity); and (**c**) Arrhenius plot of the ionic conductivity for each of the heating and cooling temperature sweeps (inset: activation energies extracted from each of these data sets). Error bars are too small to be visible on this scale. The solid lines are a guide for the eye. These independent conductivity measurements were performed between 10^-1^ Hz and 10^-7^ Hz using a Solartron 1260 impedance/gain-phase analyser. The sample pellet is placed in an impedance cell in which the sample is contacted to two stainless steel blocking electrodes. Swagelok PTFE ferrules were used to seal the impedance cell to allow measurements to be carried out in an argon atmosphere. The impedance cell could then be placed in a Lenton chamber furnace (EF 11/8B) which was monitored using a thermocouple controlled by the raspberry pi in order to measure the impedance as a function of temperature.

a

b

**Figure S6.** XRD patterns of a_m_(S-IL@ZIF-8)- 30 mins and a_m_(ZIF-8)- 20 mins. Pristine ZIF-8 was ball-milled using the same conditions as for S-IL@ZIF-8 composite. a and b are normalized and as-measured intensities, respectively.

**Figure S7.** FTIR spectra of S-IL@ZIF-8 composite (black) and of its corresponding amorphized sample ball-milled for fifteen (blue) and thirty minutes (red). Spectra resolution is 2 cm^-1^.

**Figure S8.** Arrhenius plots obtained from conductivity measurements during temperature cycling on samples having been exposed for two, six and twenty days to ambient atmosphere for: (**a**) crystalline sample, S-IL@ZIF-8 and (**b**) partially amorphized sample, a_m_(S-IL@ZIF-8)-30 mins. Note that the scale of the y-axis is the same in both figures. Error bars are in the range of four percent.

**Figure S9.** XRD patterns of amorphized ZIF-8 samples ball-milled for sixty (blue) and ninety (red) minutes.


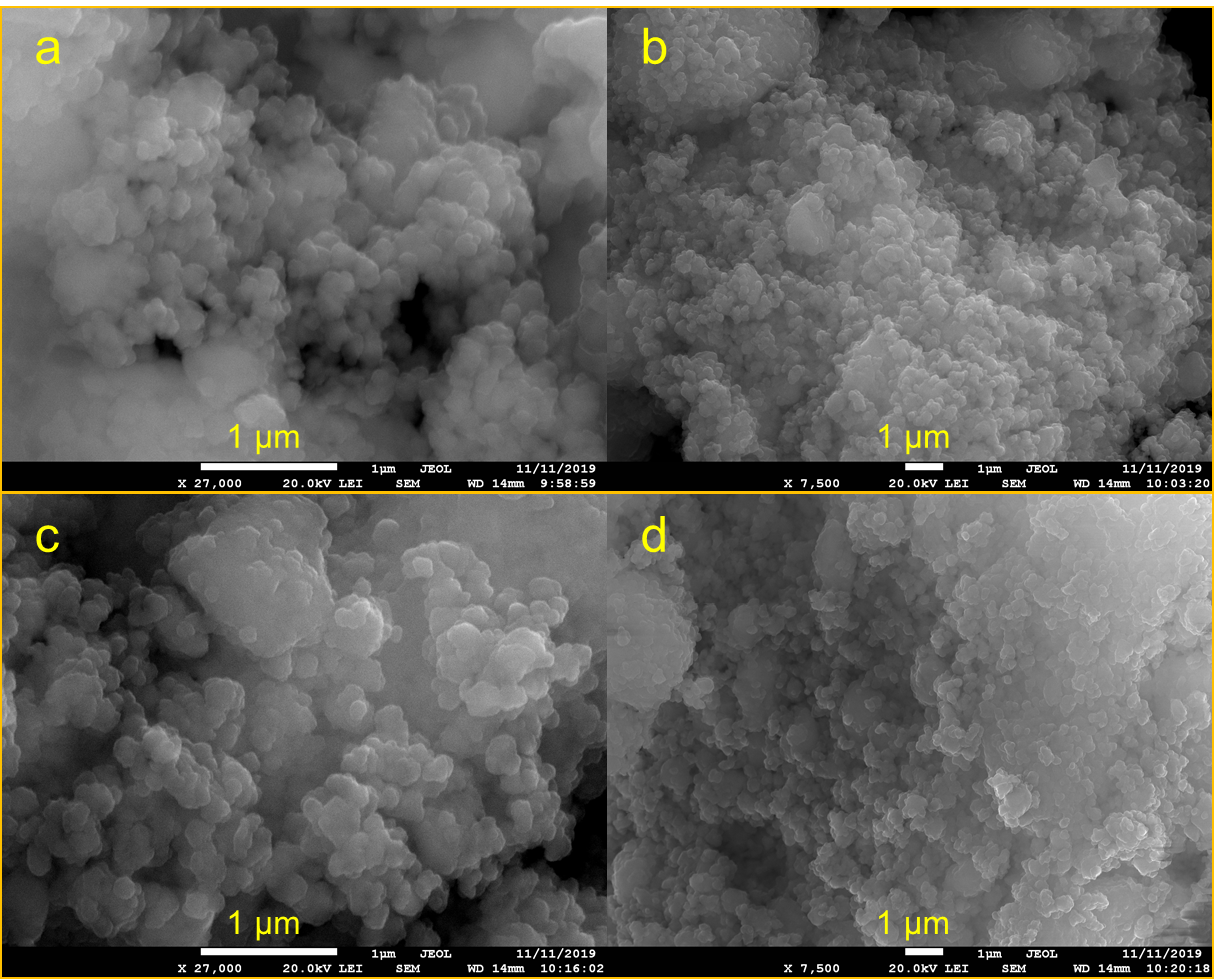


**Figure S10.** SEM images of (**a, b**) a_m_(ZIF-8)-60 mins and (**c, d**) a_m_(ZIF-8)-90 mins.
